# Novel Combination of Erythropoietin and Romiplostim to Treat Chemotherapy-Induced Anemia and Thrombocytopenia via Pharmacodynamic Interaction on Hematopoietic Stem and Progenitor Cells

**DOI:** 10.1021/acsptsci.3c00194

**Published:** 2023-11-15

**Authors:** Xiaoqing Fan, Wojciech Krzyzanski, Raymond S. M. Wong, Dongyang Liu, Xiaoyu Yan

**Affiliations:** † School of Pharmacy, Faculty of Medicine, The Chinese University of Hong Kong, Shatin, Hong Kong 999077, China SAR; ‡ Department of Pharmaceutical Sciences, The State University of New York at Buffalo, Buffalo, New York 14068, United States; § Division of Hematology, Department of Medicine and Therapeutics, Faculty of Medicine, The Chinese University of Hong Kong, Shatin, Hong Kong 999077, China SAR; ∥ Drug Clinical Trial Center, Peking University Third Hospital, Beijing 100191, China

**Keywords:** erythropoietin, romiplostim, chemotherapy-induced anemia and thrombocytopenia, pharmacodynamic interaction, hematopoietic stem and progenitor cells

## Abstract

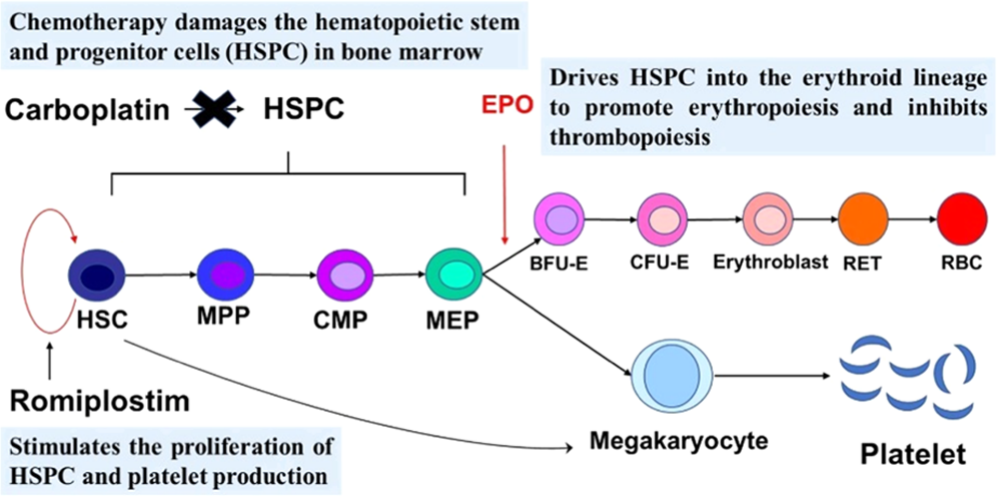

Chemotherapy-induced anemia and thrombocytopenia (CIAT) in cancer patients are often caused by the damage of hematopoietic stem and progenitor cells (HSPCs) in the bone marrow. We have previously shown that romiplostim, a thrombopoietin receptor agonist that could stimulate the expansion of HSPCs, could synergize with recombinant human erythropoietin (rHuEPO) to promote erythropoiesis in addition to stimulating platelet production, whereas rHuEPO could influence the platelet count through stem cell competition. Therefore, we hypothesize that a combination of romiplostim with rHuEPO can alleviate CIAT simultaneously, while minimizing the risk of thrombosis. In this study, we demonstrated that rHuEPO and romiplostim exhibit no stimulatory effects on the growth and invasion of LA-7 cancer cells both *in vitro* and *in vivo*. Using a rat model with carboplatin-induced anemia and thrombocytopenia, we showed that the red blood cells and hemoglobin concentration recovered faster, and the secondary thrombocytopenia was alleviated in the rHuEPO and romiplostim combination therapy groups compared with the corresponding rHuEPO monotherapy groups. The rebound phenomenon of platelets was inhibited compared with the romiplostim monotherapy group. *In vitro* study further demonstrated that romiplostim expands HSPCs and synergizes with rHuEPO to promote erythropoiesis, while rHuEPO inhibited megakaryopoiesis. Furthermore, we developed a mechanism-based pharmacokinetic-pharmacodynamic model to quantify the effects of the two drugs. This study suggests that rHuEPO and romiplostim combination therapy can treat CIAT simultaneously in rats while minimizing the risk of thrombosis, indicating that combination therapy might be superior to monotherapy in the supportive therapy of cancer patients undergoing chemotherapy.

Chemotherapy-induced anemia and thrombocytopenia (CIAT) are common complications in cancer patients undergoing myelosuppressive chemotherapy, resulting in chemotherapy cycle delay, dose reductions, and inferior overall survival.^1,2^ The pathogenesis of CIAT is complex and the damage of hematopoietic stem and progenitor cells (HSPCs) in bone marrow (BM) due to the cytotoxicity of the chemotherapeutical drugs is a major cause.^2,3^ Erythropoietin (EPO) and thrombopoietin (TPO) are the primary growth factors that stimulate red blood cells (RBCs) and platelet production, respectively. The recombinant human EPO (rHuEPO) has become one of the most effective medicines for the treatment of chemotherapy-induced anemia (CIA).^4,5^ Although currently there is no approved drug for chemotherapy-induced thrombocytopenia (CIT) management, preclinical and clinical data published to date suggested that the second-generation TPO receptor agonist (TPO-RA) romiplostim was safe and effective in increasing the platelet count in CIT, with acceptable venous thromboembolism rates.^1,6−9^

Despite the efficacy of rHuEPO for managing CIA and the promising prospect of romiplostim for managing CIT, there is no available treatment of CIAT simultaneously, which remains an important unmet need.^10^ CIT was observed in 53.6% of patients with CIA, and CIA was observed in 55.4% of patients with CIT.^10^ Approximately one-third of patients had both anemia and thrombocytopenia in the course of chemotherapy.^10^ But little attention is paid to managing CIAT simultaneously, to our knowledge, probably due to the lack of approved drugs for managing CIT currently. Moreover, about one-third of cancer patients with CIA do not respond to rHuEPO,^11^ and the risk of thromboembolic events and stimulating tumor growth raised safety controversies about using growth factors. Although the latter risk remains to be confirmed,^2,12−16^ these risks have led to researchers recommending that patients should receive the lowest rHuEPO doses possible.

Our previous studies showed that romiplostim synergized with rHuEPO to promote RBCs production while rHuEPO restores the platelet count to the normal physiological range through megakaryocyte–erythroid progenitors (MEPs) competition, thus reducing the risk of thrombosis caused by the romiplostim-induced increase in the platelet count.^17^ Therefore, the combination of EPO and romiplostim can potentially be used to treat anemia in patients who are resistant to EPO alone. As shown in Figure 1, on the one hand, romiplostim stimulates megakaryopoiesis and erythropoiesis through the MEP bifurcation pathway, but on the other hand, EPO recruits more MEPs into the erythroid lineage, reducing their MK lineage commitment. Therefore, we hypothesize that a combination of romiplostim with rHuEPO can treat CIAT at appropriate dose levels. Compared with monotherapy, combination therapy could enhance the rHuEPO response rate and reduce the dose of rHuEPO to avoid the potential off-target effect. At the same time, it could prevent thrombocytosis to minimize the risk of thrombosis.

**Figure 1:**
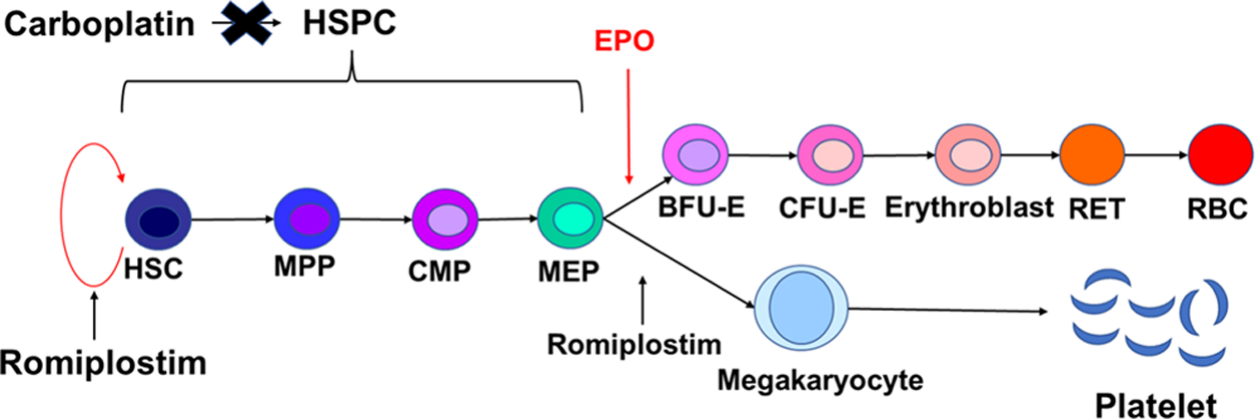
Schematic illustration for erythropoiesis and thrombopoiesis, including the effects of carboplatin, rHuEPO, and romiplostim. Carboplatin induces the damage of hematopoietic stem and progenitor cells (HSPCs), rHuEPO stimulates the differentiation of HSPCs into the erythroid lineage, while the romiplostim stimulates the self-renewal of HSPCs and its differentiation into megakaryocyte.

In this study, we evaluated the effects of rHuEPO and romiplostim on LA-7 tumor cell growth and invasion both *in vitro* and *in vivo*. Using LA-7-induced orthotopic rat model with carboplatin-induced anemia and thrombocytopenia, we investigated the pharmacokinetics (PK) and pharmacodynamics (PD) of rHuEPO and romiplostim as monotherapy and combination therapy to test our hypothesis. *In vitro* study of HSPCs was further conducted to elucidate the mechanism. A mechanism-based PK-PD model was established to quantify the effects of rHuEPO and romiplostim on the hematological response. The results would provide the rationale for the direct translation of this novel combination therapy approach for treating CIAT simultaneously in cancer patients undergoing chemotherapy while also introducing new mechanistic findings that relate to chemotherapy with hematopoiesis.

## Materials and Methods

### Reagents

RHuEPO (EPOGEN 20,000 units/mL) was purchased from Amgen, Inc. (CA, USA). Romiplostim (Romiplate, 250 μg) was obtained from Kyowa Kirin (Hong Kong). Carboplatin was purchased from MedChemExpress (NJ, USA).

### Cell Culture

The LA-7 (also called CRL-2283), a rat mammary gland tumor cell line with stem cell characteristics, was purchased from the American Type Culture Collection (ATCC, VA, USA). The cells were cultured in high-glucose (4.5 g/L) Dulbecco’s modified Eagle’s medium (DMEM) supplemented with 5% fetal bovine serum (FBS), and 100 U/mL penicillin/streptomycin at 37 °C with 5% CO_2_ in a cell incubator (Thermo Fisher).

### *In Vitro* Cytotoxicity Study

Cytotoxicity of rHuEPO and romiplostim on LA-7 cells was assessed by employing the 3-(4,5-dimethylthiazol-2-yl)-2,5-diphenyltetrazolium bromide (MTT) cell viability assay. Briefly, LA-7 cells were cultured in 96-well plates for 24 h at a seeding density of 5 × 10^4^ cells/cm^2^. The experiments were initiated by aspirating the culture medium in each well and incubating the cells with varying concentrations of rHuEPO (0.01, 0.05, 0.1, 0.2, 0.5, 1, 2, 5, 10, 20, 50, 100 IU/mL, diluted by DMEM), romiplostim (0.01, 0.02, 0.05, 0.1, 0.2, 0.5, 1, 2, 5, 10, 20, 50, 100 ng/mL, diluted by DMEM), or an equal volume of DMEM (vehicle control) for 24 or 48 h at 37 °C in a 5% CO_2_ incubator. An aliquot in each well was aspirated, and the cells were then incubated for a further 4 h with the MTT solution. The medium was removed and 150 μL of dimethyl sulfoxide was added to each well to dissolve the purple formazan product, and the plates were incubated at 37 °C for another 10 min to remove air bubbles. Cell viability was defined as the ratio of absorbance (treated to untreated cells) at 490 nm (Bio-Rad Laboratories).

Carboplatin is a second-generation platinum compound that has strong toxicity to BM, which can be used to induce CIAT simultaneously in rats.^18^ To evaluate the effect of rHuEPO and romiplostim on carboplatin-induced cytotoxicity on LA-7 cells, an MTT assay was performed as described above. The cells were exposed to carboplatin (7.8125, 15.625, 31.25, 62.5, 125, 250, 500, 1000 μg/mL) for 48 h in the presence or absence of the rHuEPO (1 IU/mL), romiplostim (4 ng/mL), or rHuEPO (1 IU/mL) + romiplostim (4 ng/mL) at 37 °C in a 5% CO_2_ incubator.

### Cell Invasion Study

To evaluate the effect of rHuEPO and/or romiplostim on LA-7 cell invasion, the cell invasion assay was performed using the Cell Invasion Assay Kit (ab235884; Abcam plc.; Hong Kong) according to the manufacturer’s instructions. Briefly, the LA-7 cells were starved for 24 h in serum-free media before the assay and then seeded in the top chamber. 5 × 10^4^ cells/50 μL cell suspension was added to each well of the top chamber. 200 μL of medium per well containing positive invasion inducer (provided by the kit), rHuEPO (0.1, 1, 50 IU/mL), romiplostim (0.1, 1, 10 ng/mL), and rHuEPO + romiplostim (1 IU/mL + 1 mg/mL, 1 IU/mL + 10 mg/mL, 50 IU/mL + 1 mg/mL, 50 IU/mL + 10 mg/mL) were added to the bottom chamber. Following 48 h of the incubation period, the cells that migrated to the bottom chamber were stained with a cell invasion dye. Then, stained cells were quantified by fluorescence (Em/Ex 530/590). And the cell invasion percentage was defined as the ratio of cells in the lower chamber to the total cells added to the top chamber.^19^

### Animal Model

Animal experiments were conducted upon the approval of the Animal Experimentation Ethics Committee of The Chinese University of Hong Kong (CUHK, Reference Number 21-304-MIS). Female Sprague–Dawley rats of weight from 160 to 200 g (Provided by the Laboratory Animal Services Centre at CUHK at least 1 week before the experiment to acclimate) were kept under humidity- and temperature-controlled environment in a 12 h light/darkness cycle (2–3 rats per cage) and had access to water and food ad libitum. Rats were fed standard food supplemented with 1% (w/w) carbonyl iron (Test Diet). For tumor induction, rats were injected subcutaneously into the mammary fat pad (right flank) with 1.5 × 10^7^ LA-7 cells suspended in 0.3 mL of phosphate-buffered saline (PBS; Sigma-Aldrich) using a tuberculin syringe and 26G needle. Injection of cells was performed under isoflurane (0.4%) anesthesia, and cells (P#6) were used within 2 h of preparation. Tumors were measured manually beginning 2 days post mammary tumor induction using a caliper. The tumor volume (mm^3^) was calculated as follows: length × width^2^/2.^20^ Rats with breast cancer received a single dose of carboplatin (60 mg/kg) through intravenous (IV) injection in the tail vein 4 days post tumor cells injection^18^ to induce anemia and thrombocytopenia, the tumor control group received saline only.

### PK and PD of rHuEPO, and Romiplostim in CIAT Rats

The PK and PD study design was based on previous experiments.^17^ There were 9 groups, including the tumor control group, carboplatin-treated control group, rHuEPO monotherapy groups (three dose levels, including 100, 450, and 1350 IU/kg), romiplostim monotherapy group (30 μg/kg), and rHuEPO plus romiplostim combination therapy groups (100 IU/kg + 30 μg/kg, 450 IU/kg + 30 μg/kg, and 1350 IU/kg + 30 μg/kg). For the PK study of rHuEPO and romiplostim, rats with CIAT received rHuEPO 100, 450, or 1350 IU/kg (thrice-weekly for 2 weeks) through IV injection in the tail vein as monotherapy or received romiplostim 30 μg/kg monotherapy (once weekly for 2 weeks) through subcutaneous injection on the upper back area or a combination of the two on day 8 (4 days post carboplatin injection). Each treatment group (*n* = 6) was divided into two subsets (A and B), and each subset contained three rats. Blood samples were collected from two subsets of rats through the tail vein in a rotating manner, and only peak and trough concentration–time points were collected to minimize total blood loss. Specifically, for rats treated with rHuEPO, blood samples were drawn from subset A at 5 min and from subset B at 32 h after the dose. The total volume of blood drawn from each rat per 72 h was 160 ± 40 μL, with 80 ± 20 μL drawn at each time point. For rats treated with romiplostim, blood was collected from subset A at 16 h and from subset B at 72 h postdose. For rats treated with rHuEPO plus romiplostim, blood was collected from subset A at predose, 16 h, and 72 h postdose. While for subset B, blood was collected at 5 min and 32 h postdose. PK profiles were obtained on days 8 and 15.

The PD markers include RBCs, hemoglobin (Hgb), and platelet. Blood samples for PD analysis were drawn on days 0, 4, 8, 10, 12, 15, 17, 19, 22, 24, 26, 29, 31, 33, 36, and 38, until day 40, on which the value of PD markers returned to baseline. Before sampling, rats were anesthetized with 0.4% isoflurane. The PD markers were measured using a BC2800VET Hematology Analyzer (Mindray).

### Flow Cytometry

To evaluate the effect of rHuEPO and romiplostim on the BM erythroid cells, flow cytometric analyses were performed using a flow cytometer (BD LSR Fortessa) following the protocol published before.^17,21^ Briefly, the rats were sacrificed by exsanguination under anesthesia at the end of the observation (day 40). BM was gently extracted from the femurs with a 21-gauge needle containing 4 mL of Iscove’s Modified Dulbecco’s Medium supplemented with 1% penicillin/streptomycin. The freshly isolated BM cells were stained with trypan blue and counted using a Countess 3 automated cell counter (Invitrogen). Then, 10^6^ cells were incubated with 0.03 μg of biotin-conjugated antirat HIS49 (erythroid cells) antibody (BD Biosciences) in 100 μL PBS/0.5% BSA with the presence of rat IgG for 30 min. After three washes, the cells were further incubated with APC-conjugated streptavidin (BD Biosciences), and 0.03 μg of PE-conjugated antirat CD71 was used for staining CD71 concurrently with the secondary stain. The forward scatter parameter was used to evaluate the erythroid maturation level.

### Colony-Forming Unit Assay

For the colony-forming unit (CFU) assays to evaluate the effect of rHuEPO and romiplostim on HSPCs, the experiment procedure has been previously described.^17^ Specifically, multipotent HSPCs were collected from normal rat BM by using mouse antirat CD90 (Thy-1.1; BD Bioscience) and the EasySep Rat Custom Positive Selection Kit (STEMCELL) according to the manufacturer’s instructions. Enriched HSPCs were initially cultured in StemSpan Serum-Free Expansion Medium at a density of 1 × 10^5^ cells/mL in T-25 flasks (Corning) and incubated at 37 °C with 5% CO_2_ in a cell incubator. Stem cell factor (SCF, 100 ng/mL) (R&D Systems), fms-related tyrosine kinase 3 ligand (FL, 100 ng/mL) (STEMCELL), and different concentrations of romiplostim were added to induce the proliferation of the HSPCs (first step: day 0–5). The viable cells were stained with trypan blue and were counted on day 5. Then, to evaluate the effect of rHuEPO on differentiation of the HSPCs (second step: day 6–10), cytokines including 100 ng/mL of SCF, 100 ng/mL of FL, 15 ng/mL of rat recombinant granulocyte-macrophage colony-stimulating factor (GM-CSF, STEMCELL), 20 ng/mL of interleukin (IL-3, R&D Systems), and 2 or 4 ng/mL romiplostim were added, in addition to different concentrations of rHuEPO to induce differentiation into MK or erythroid lineage, respectively. MethoCult (STEMCELL) was used for the growth of BFU-E and CFU-E according to the manufacturer’s instructions. For the CFU-MK assay, acetylcholinesterase, a relatively specific marker of MK, was stained to detect CFU-MK colonies.^22,23^ BFU-E, CFU-E, and CFU-MK were counted using a bright-field Eclipse Ti microscope and NIS-Elements Color Cam Ver. 4.00 (Nikon). To link the proliferation and the differentiation, the number of CFUs in the second step is multiplied by the absolute number of HSPCs in different concentrations of romiplostim-treated groups in the first step to compare the changes in total CFU number.

### Bioanalytical Methods

Serum concentrations of romiplostim were determined by a sandwich enzyme-linked immunosorbent assay (ELISA) developed before.^17^ Briefly, the AffiniPure mouse antihuman IgG, Fcγ fragment specific, and the peroxidase AffiniPure mouse antihuman IgG, Fcγ fragment specific (Jackson Immuno Research Laboratories) were employed as the capture antibody and the detection antibody, respectively. The concentration of romiplostim captured in the antibody sandwich was measured spectrophotometrically at 450 nm using a Benchmark microtiter plate reader (Bio-Rad Laboratories). Fitted curve of romiplostim showed good linearity (*r*^2^ ≥ 0.99) in the calibration concentration range of 0.25–25 ng/mL. A commercial ELISA kit (R&D Systems) was used to measure the serum concentrations of rHuEPO (assay range 2.5–200 mIU/mL). This assay is specific for rHuEPO and did not measure endogenous EPO. Thus, it was assumed that the endogenous EPO is negligible.^24^ The rat vascular endothelial growth factor (VEGF) levels in the serum were measured using a commercially available ELISA kit (Abcam plc.; Hong Kong). The assays were performed according to the manufacturer’s instructions. The collected serum was stored at −20 °C until the assay was performed.

### Development of Mechanism-Based PK-PD Modeling

To quantify the erythropoietic and thrombopoietic effects of EPO and romiplostim in rats with CIAT, a mechanism-based PK-PD model was developed.

#### PK Models for Carboplatin, rHuEPO, and Romiplostim

The carboplatin PK data in rats following single i.v. dose (60 mg/kg) were fitted to a three-compartment model,^18^ while a two-compartment disposition model and a one-compartment model were used to describe the time course of rHuEPO and romiplostim, respectively.^17^ The differential equations are as follows (eqs 1–7):
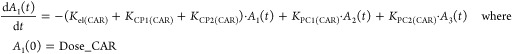
1
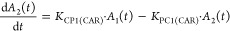
2
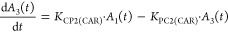
3
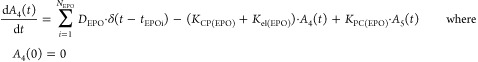
4
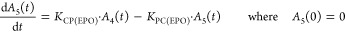
5
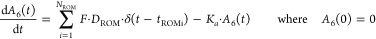
6
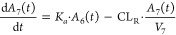
7where *A*_1_(*t*), *A*_2_(*t*), and *A*_3_(*t*) are the amounts of carboplatin in the central, peripheral 1 and 2 compartments, respectively. *A*_4_(*t*) and *A*_5_(*t*) are the amounts of rHuEPO in the central and peripheral compartments, respectively. *A*_6_(*t*) and *A*_7_(*t*) are the amounts of romiplostim in the deposition and central compartments, respectively. Initial conditions for *A*_2_, *A*_3_, and *A*_7_ were zero. The symbol *D*_EPO_·δ(*t* – *t*_EPOi_) indicates a bolus input of dose *D*_EPO_ at time *t*_EPOi_. CL_R_ and *k*_a_ represent the apparent clearance and first-order absorption rates of romiplostim, respectively. *K*_el(CAR)_ is the linear elimination rate constant of carboplatin, and *K*_CP1(CAR)_, *K*_CP2(CAR)_, *K*_PC1(CAR)_, and *K*_PC2(CAR)_ are the intercompartmental rate constants for tissue distribution of carboplatin, respectively. *V*_4_ and *V*_5_ denote the volumes of the central and peripheral compartments of rHuEPO, respectively. *K*_el(EPO)_ is the linear elimination rate constant of rHuEPO. *K*_CP(EPO)_ and *K*_PC(EPO)_ are the intercompartmental rate constants for tissue distribution of rHuEPO, respectively, which are parametrized in terms of the clearance and volumes of distribution of rHuEPO as follows:
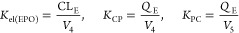
8where *Q*_E_ is the intercompartmental flow of *A*_4_ and *A*_5_. The concentration of EPO to maintain erythropoiesis can be written as:
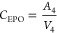
9

#### PD Model

The PD model was described by using a catenary-lifetime-based indirect response model, which was modified from our previous publication.

The hematopoietic toxicity of carboplatin in BM is to reduce the proliferation and differentiation rate or induce cell apoptosis.^18,25−27^ The HSPCs with a high capacity for proliferation were considered to be the primary cellular target. The stimulatory effect of romiplostim is on the production rate of HSPCs and the differentiation of HSPCs into BFU-E cells is controlled by the processes with the first-order rate constant KE, which can be stimulated by rHuEPO,^17^ as follows (eq 14):
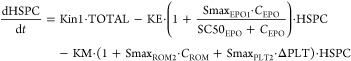
10
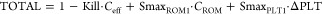
11
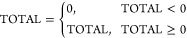
12where Kin1 is a zero-order rate constant for producing the HSPCs; *C*_ROM_ and *C*_EPO_ are the serum concentrations of romiplostim and rHuEPO at time *t*, respectively; Smax_ROM1_ and Smax_ROM2_ are the slope parameters for linear stimulation of romiplostim on Kin1 and differentiation of HSPCs into MK, respectively; Smax_EPO_ is the maximal stimulus of rHuEPO on differentiation of HSPCs into BFUE; and SC50_EPO_ is the concentration of rHuEPO that induces a half-maximum effect. HSPCs differentiate into erythroid and MK lineages according to the first-order rate constant KE and KM, respectively. ΔPLT represents changes from the baseline of platelet (ΔPLT = PLT0 – PLT_*t*_). The linear stimulatory functions Smax_PLT1_·ΔPLT and Smax_PLT2_·ΔPLT represent the feedback mechanism of the thrombopoiesis process, which was directly used as a regulator for the negative feedback loop due to the lack of endogenous TPO PK data. This feedback stimulation on HSPCs could explain the subsequent increase in platelets in the carboplatin-treated control group.^28^

Kill is the slope parameter for linear inhibition of carboplatin on Kin1 and differentiation of HSPCs into BFUE and MK; *C*_eff_ is the effect site concentration of carboplatin. The effect compartment link model was used to describe the time displacement between the measured concentration and the observed effect of carboplatin, which occurs due to a delayed distribution between the drug concentration in plasma and the effect site.^29^ The relationship between the concentration at the effect compartment and the serum concentration *C*_CAR_ is expressed by the differential equation as below:
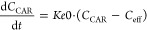
13

*Ke*0 is the equilibration rate constant that determines the concentration equilibrium between the plasma and the effect compartment.

The stimulatory effect of rHuEPO is added to the differentiation of HSPCs into BFU-E, which could explain the depletion of the platelet.^17^
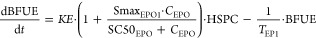
14
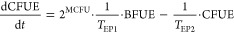
15
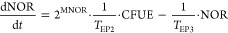
16
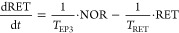
17
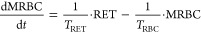
18where 2^MCFU^ and 2^MNOR^ reflect that one BFU-E and CFU-E cell can produce 2^MCFU^ CFU-E cells and 2^MNOR^ NORs, respectively.^30^*T*_EP_, *T*_RET_, and *T*_RBC_ are the mean lifespans of precursors, RETs, and mature RBCs, respectively.^31^*T*_EP_ was assumed to be equal to *T*_RET_, which further reduced the number of model parameters. HGB concentrations were derived from the mass of mature RBCs (MRBC) and RETs:
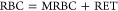
19
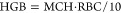
20where MCH is the mean corpuscular HGB, which was estimated directly from the data.^30^ The numerator 10 converts the MCH unit to pg/cell.^31^

For platelet production, romiplostim stimulates the production of HSPCs and their differentiation into MK1. Carboplatin stimulates the apoptosis of MK.^25^ The differential equations are as follows (eqs 23–25):
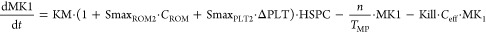
21
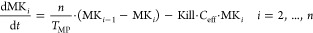
22

A series of aging compartments (MK*n*, *n* = 10) denoted the MK precursor cells in BM, with the first-order transition rates *n*/TMP.

Similarly, PLT*n* (*n* = 10) represents the platelets in PB with the transition rates *n*PLT/TPLT:
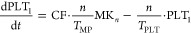
23
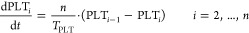
24where *T*_MP_ and *T*_PLP_ denote the mean life spans of precursor cells and platelets, respectively, and CF represents the conversion factor equal to the average number of platelets produced by an MK and was fixed at 4000.^32^ The platelets were modeled as the sum of the platelet counts in each PLT compartment:
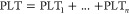
25

The secondary parameters and baseline equations defined by the steady-state value can be used to reduce the number of model parameters as follows:
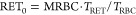
26
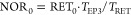
27
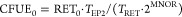
28
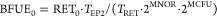
29
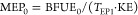
30
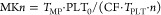
31
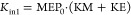
32
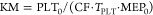
33

Considering that only a small sample size (*n* = 6) is available for sparse-sample study, a naïve pooled data approach was used, in which all data from all individuals are considered as arising from one unique individual.

The residual variability in RBC, PLT, and HGB was added separately. A combined (proportional plus additive) model of residual error was applied and described as:
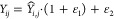
34where *Y_ij_* is the observation of individual *i* at time *t_j_*, *Ŷ*_*ij*_ is the corresponding model prediction, ε_1_ is the proportional error, and ε_2_ is the additive error, both of which are assumed to be independent and normally distributed random variables, with a mean of zero and a variance of σ_1_^2^ and σ_2_^2^, respectively.

### Model Evaluation and Model-Based Simulations

Model selection and evaluation were based on the objective function value, parameter precision, and visual inspection of the graphical diagnostics. The diagnostic plots were plotted by R as the initial visualized check that include observed value versus population predicted value and individual predicted value, conditional weighted residual (CWRES) versus population predicted value, and CWRES versus time. The final model was further evaluated by simulating the time course of PD values using the parameters estimated in the final model. The observed data were plotted overlaid with the simulated data. To investigate the drugs’ effects on HSPC, BFUE, and MK1 in BM, model-based simulations using the final model were conducted.

### Software and Statistical Analyses

The experiments were carried out in a randomized manner. PK/PD model analysis was performed using NONMEM 7.5 (Icon Development Solutions, Ellicott City, MD). The use of NONMEM was facilitated by Perl-speaks-NONMEM (version 4.9.6, http://psn.sourceforge.net/docs.php). The ordinary differential equations were solved by ADVAN14 subroutine, and the first-order conditional estimation method with interaction (FOCEI) algorithm was used for parameter estimation. Comparisons were performed using Student’s unpaired *t* test, one-way ANOVA with Dunnett’s multiple comparisons test, or two-way ANOVA with Dunnett’s multiple comparisons test, with *p* < 0.05 considered to indicate statistically significant differences. Comparisons and plotting were performed using the R program (version 4.1.1, www.r-project.org) or GraphPad Prism (version 9.5.1).

## Results

### EPO and Romiplostim Exhibit No Stimulatory Effects on the Growth and Invasion of LA-7 Breast Cancer Cells *In Vitro*

We first studied the effects of EPO and romiplostim on the growth of LA-7 cells using the MTT assay to evaluate the safety of using EPO and romiplostim *in vivo* because LA-7 cells express EPO receptors.^33^ The cell viability remains constant after 24 or 48 h exposure to different concentrations of rHuEPO (0.01–100 IU/mL) and romiplostim (0.01–100 ng/mL), which indicated that rHuEPO and romiplostim exhibit no stimulatory effects on the growth of LA-7 breast cancer cells *in vitro* (Figure S1A,B).

Carboplatin is a representative chemotherapeutic drug with strong BM cytotoxicity. To evaluate whether rHuEPO and romiplostim could affect carboplatin-induced cytotoxicity on tumor cells, the carboplatin cytotoxicity (7.8 −1000 μg/mL) was estimated with or without simultaneous exposure to rHuEPO and romiplostim for 48 h. The LA-7 cell viability was decreased with the increasing concentration of carboplatin, after the treatment of the rHuEPO (1 IU/mL), romiplostim (4 ng/mL), or rHuEPO (1 IU/mL) + romiplostim (4 ng/mL), there was no significant change in the cytotoxicity of carboplatin, with the cell viability curves in parallel with the carboplatin control group (Figure S1C).

Cell invasion assays were performed to investigate whether rHuEPO and romiplostim could induce cancer cell invasion. The positive invasion inducer treatment significantly increased LA-7 cell invasion rate (*p* < 0.001), whereas rHuEPO (0.1, 1, 50 IU/mL), romiplostim (0.1, 1, 10 ng/mL), and rHuEPO + romiplostim (1 IU/mL + 1 mg/mL, 1 IU/mL + 10 mg/mL, 50 IU/mL + 1 mg/mL, 50 IU/mL + 10 mg/mL) exhibit no significant influence on LA-7 cell invasion rate (Figure S1D).

### Combination Treatment Promotes RBCs Production Synergistically and Maintains Platelet Count to a Normal Range

**Figure 2:**
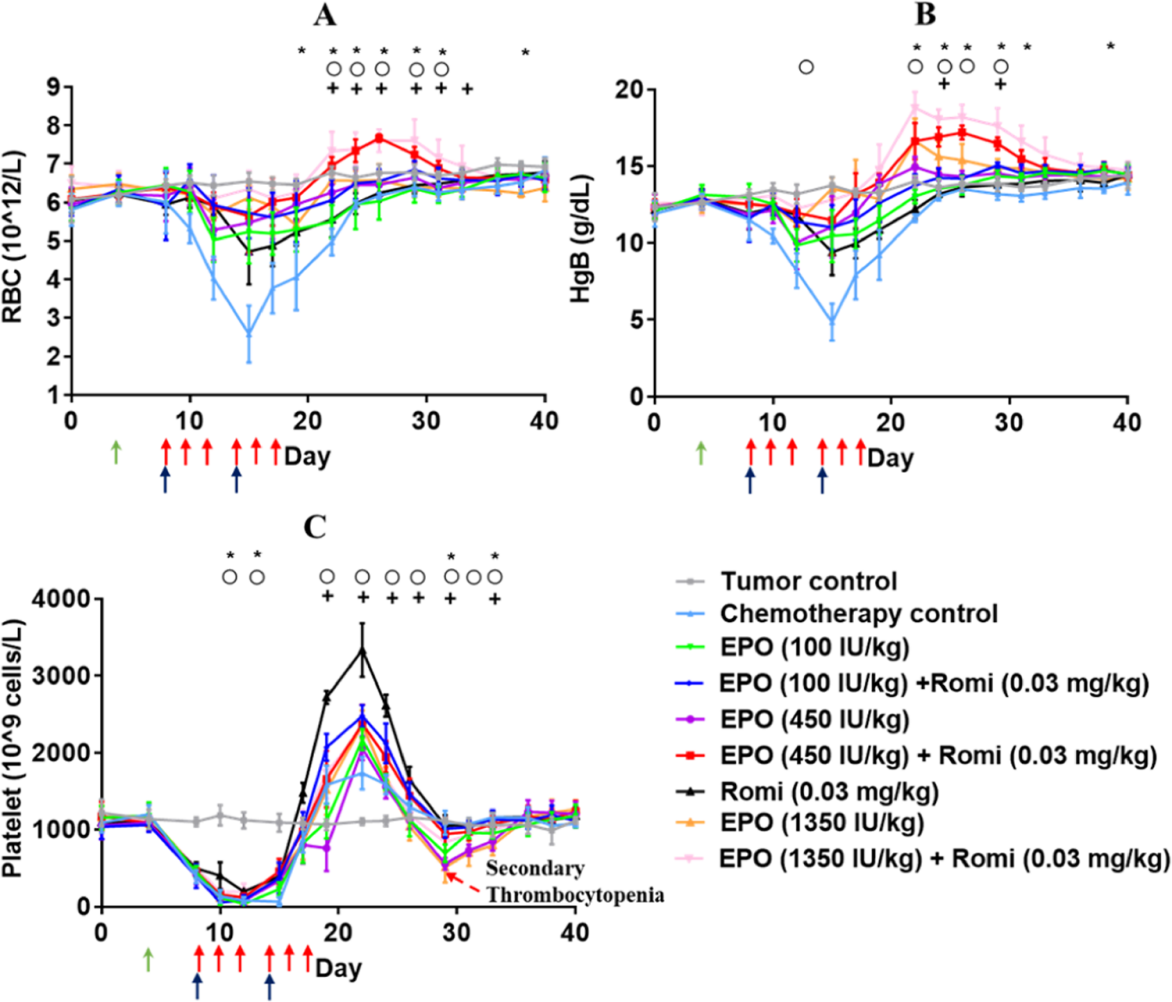
Pharmacodynamics of rHuEPO (100, 450, and 1350 IU/kg, TIW for 2 weeks) and Romiplostim (0.03 mg/kg, QW for 2 weeks) as monotherapy and combination therapy in peripheral blood. (A) Mean RBC time course profile. (B) Mean Hgb time course profile. (C) Mean platelet time course profile. The arrows represent the dosing event of carboplatin (green), rHuEPO (red), and romiplostim (black). Data were expressed as mean ± standard deviation (*n* = 6). The symbols above the lines indicate days of statistically significant differences between the following rHuEPO monotherapy groups and the corresponding rHuEPO plus romiplostim combination therapy groups: + = 100 IU/kg; o = 450 IU/kg; * = 1350 IU/kg; (*p* < 0.05, Student’s unpaired *t* test).

To induce CIAT in cancer rats, carboplatin (60 mg/kg) was IV injected through the tail vein 4 days post tumor cells injection. As shown in Figure 2, the carboplatin-treated cancer rats exhibited progressive anemia and thrombocytopenia. Compared with the tumor control group, RBCs, Hgb and platelet decreased and reached a nadir by day 15 after administration of carboplatin and returned to the baseline by day 29 for RBCs and Hgb (Figure 2A,2B), and by day 19 for platelet (Figure 2C). One rat died due to severe anemia on day 15 (Hgb = 2.8 g/dL). There was a rebound phenomenon for platelets, and the rebound peak occurred by day 22, followed by a slow return to baseline levels.

Then, the effects of rHuEPO on RBCs and platelet production in cancer rats with CIAT were studied. As shown in Figure 2, the RBCs and Hgb concentration recovered faster in rHuEPO monotherapy groups compared to carboplatin-treated rats, which reached a nadir by day 12 and then increased in a dose-dependent manner. The platelet counts also reached a nadir by day 12 followed by the rebound phenomenon. However, the platelet decreased below the baseline value and reached a second nadir on day 29 in a dose-dependent manner, which was 36.4, 49.1, and 53.1% below the baseline value, respectively. These results indicated that although rHuEPO could promote erythropoiesis to improve CIA, intensive rHuEPO treatment could inhibit platelet production and induce secondary thrombocytopenia (reduced platelet counts below the baseline 2 weeks after cessation of the romiplostim treatment, as shown in Figure 2C), increasing the risk of bleeding.

We next investigated the effects of romiplostim (30 μg/kg) as a monotherapy and in combination with rHuEPO *in vivo*. As shown in Figure 2, romiplostim monotherapy had a strong stimulatory effect on platelet production (Figure 2C), and a moderate effect on RBCs and Hgb concentration, compared with the chemotherapy control and rHuEPO monotherapy groups. Notably, the RBCs and Hgb recovered faster in the rHuEPO plus romiplostim combination groups compared with corresponding rHuEPO monotherapy groups, which peaked on day 29 and then return to baseline levels. The results suggested a synergistic effect of rHuEPO and romiplostim on erythropoiesis and are consistent with our previous work in CKD rats.^17^ Importantly, the rebound peak of platelet in the combination therapy groups was inhibited compared with the romiplostim monotherapy group, while the secondary thrombocytopenia was alleviated compared with the rHuEPO monotherapy groups. These results, together with the hematopoiesis model (Figure 1), support our hypothesis that romiplostim could improve CIT and synergize with rHuEPO to improve CIA simultaneously. At the same time, the combination of rHuEPO could help to minimize the fluctuation of platelets and maintain their count to the normal physiological range, subsequently reducing the risk of thrombocytosis and secondary thrombocytopenia.

### Combination Treatment Restores BM Total Cell Number and Erythroid Precursors Synergistically

**Figure 3:**
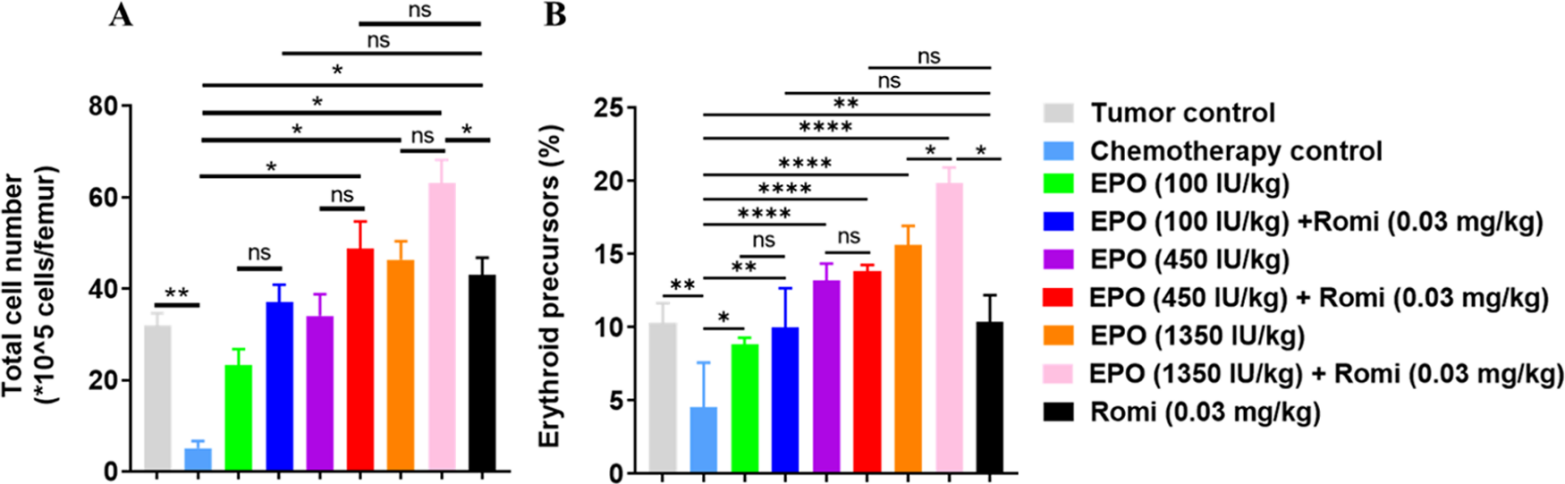
(A) Bone marrow total cell number at end point (day 40). (B) Bone marrow erythroid precursors percentage at end point (day 40). Data were expressed as mean ± standard deviation (*n* = 3). *p < 0.05. **p < 0.01, ****p < 0.0001. One-way ANOVA with Dunnett’s multiple comparisons test for bone marrow total cell number and erythroid precursors percentage.

To investigate the effects of rHuEPO and romiplostim on the BM, the BM total cell number and the erythroid precursor cells percentage were measured at the end of the study (Day 40). It can be seen that the total BM cell number decreased significantly in the chemotherapy control group (*p* < 0.01), while the cell number recovered in the treatment groups in a dose-dependent trend, and the combination therapy has better efficacy than the corresponding EPO monotherapy groups (Figure 3A). Similarly, the erythroid precursor percentage also decreased significantly in the carboplatin-treated group compared with the tumor control group. After being administered with EPO and romiplostim as monotherapy or combination therapy, the erythroid precursors’ percentage was restored in a dose-dependent manner, and the combination therapy has better efficacy than the corresponding EPO monotherapy groups (Figure 3B). These results further demonstrated that romiplostim could expand HSPCs, thus synergizing with EPO to restore erythroid precursors and promote erythropoiesis.

### Effect of Combination Therapy Is Due to PD Interaction Instead of PK Interaction

**Figure 4:**
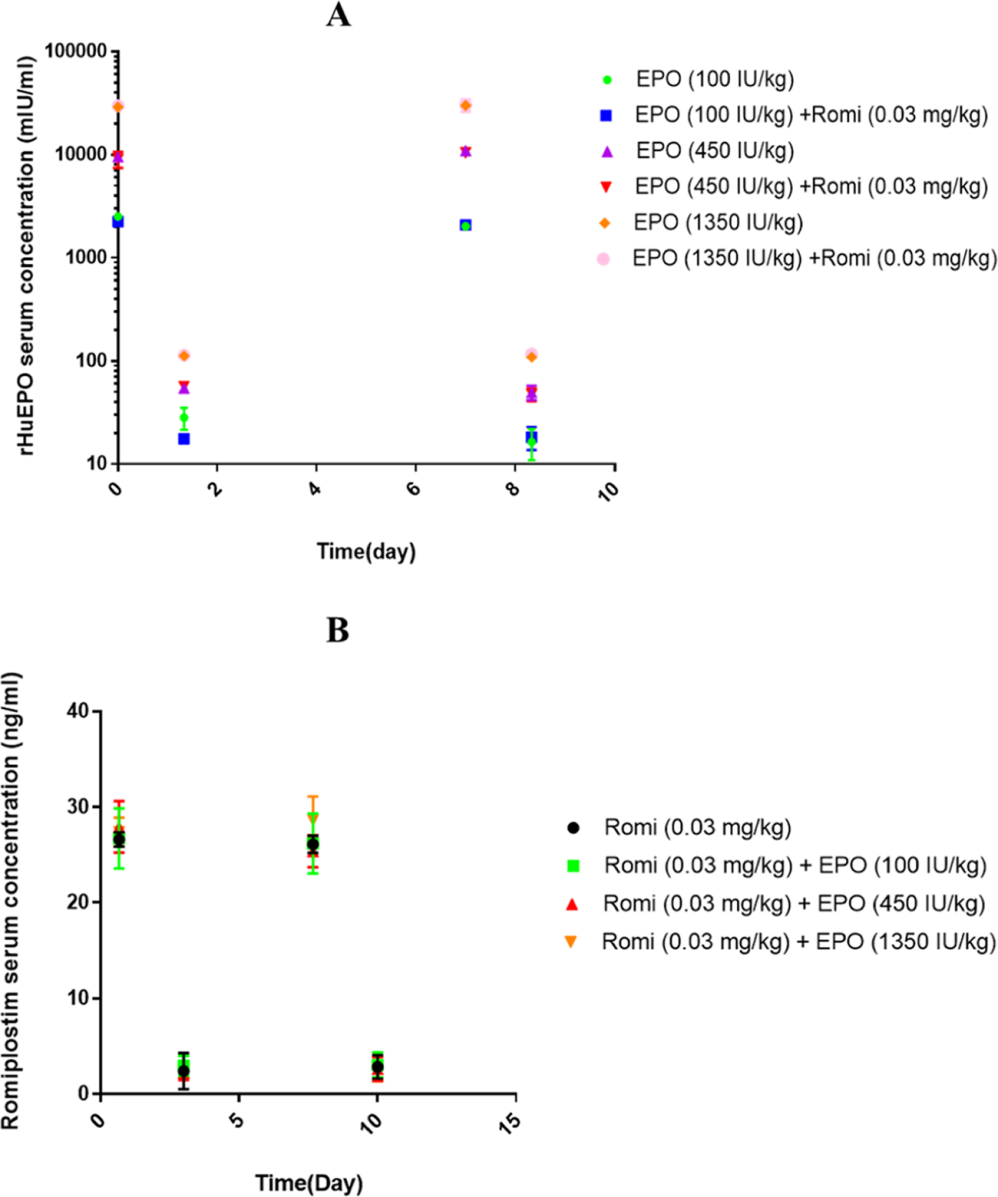
RHuEPO serum postdose concentrations during multiple dosing regimens of rHuEPO in the presence or absence of romiplostim (A) and romiplostim serum postdose concentrations during multiple dosing regimens in the presence or absence of rHuEPO (B). The symbols depict the mean profile with standard deviation error bars (*n* = 6).

PK interaction could change the drug concentration and lead to the subsequent change in the drug effect.^34^ To investigate whether the observed effects in the combination therapy group are due to the PK interaction, we studied the PK of rHuEPO and romiplostim in the treatment groups. The peak and trough drug concentrations are shown in Figure 4A,4B. It can be seen that there is no significant difference in the PK profiles of rHuEPO (Figure 4A) and romiplostim (Figure 4B) between the monotherapy and combination therapy groups. These results suggested that there was no PK interaction between rHuEPO and romiplostim in the combination groups at the dosing regimens adopted for our cancer rats with CIAT. This further indicated that the effects observed in the combination group were due to PD interaction rather than PK interaction.

### Romiplostim Expands HSPCs, While rHuEPO Promotes Erythropoiesis and Inhibits Megakaryopoiesis

**Figure 5:**
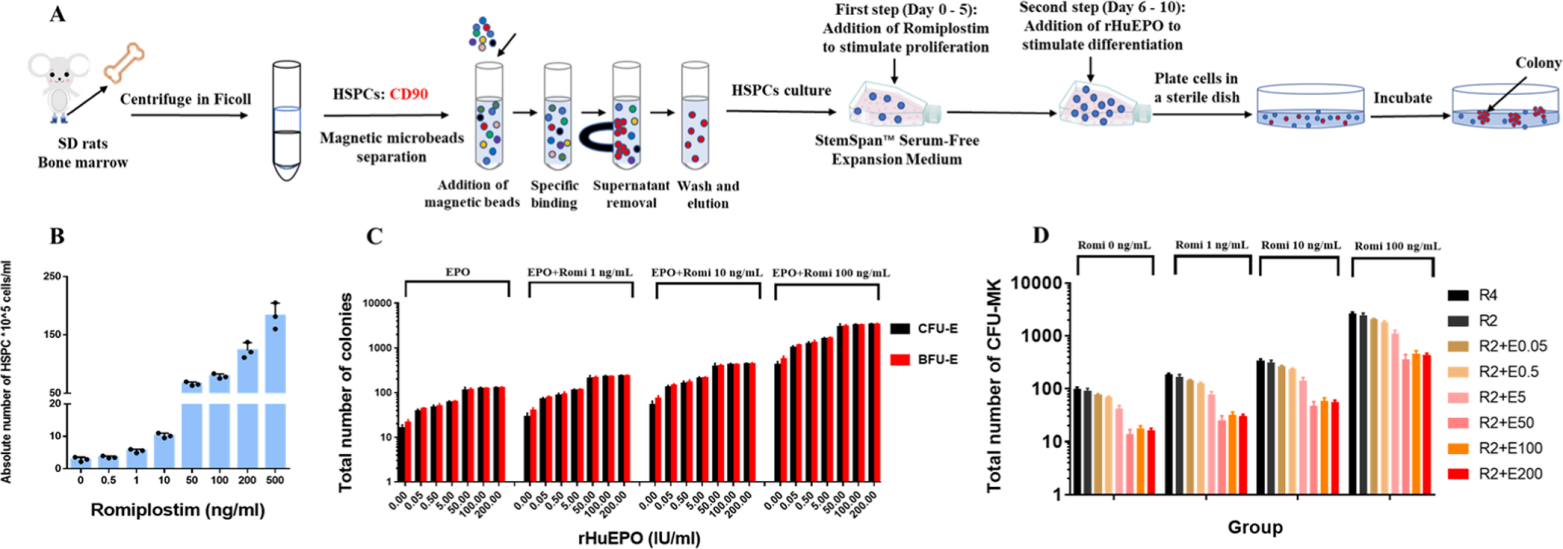
Romiplostim expands HSPCs and synergizes with rHuEPO to promote erythropoiesis, while rHuEPO drives progenitors toward the erythroid fate to inhibit thrombopoiesis through progenitor cell competition. (A) Schematic representation of the HSPCs proliferation and differentiation *in vitro* study design. (B) Absolute numbers of total HSPCs with different concentrations of romiplostim on day 5. (C) Total number of colonies (BFU-E and CFU-E) at different concentrations of rHuEPO on day 10 following 5 days of incubation (day 6–10). The first column (EPO) is the original experimental data, while the last three columns are the number of CFUs multiplied by the absolute number of HSPCs in different concentrations of romiplostim-treated groups in the first step to compare the change in total CFU number. (D) Total number of colonies (CFU-MK) at different concentrations of rHuEPO on day 10 following 5 days of incubation (day 6–10) in indicated concentrations of romiplostim. The first column (Romi 0 ng/mL) is the original experimental data, while the last three columns are the number of CFUs multiplied by the absolute number of HSPCs in different concentrations of romiplostim-treated groups in the first step to compare the change in total CFU number. R4 = romiplostim 4 ng/mL, R2 = romiplostim 2 ng/mL, R2 + E0.05 = romiplostim 2 ng/mL + rHuEPO 0.05 IU/mL, R2 + E0.5 = romiplostim 2 ng/mL+ rHuEPO 0.5 IU/mL, R2 + E5 = romiplostim 2 ng/mL+ rHuEPO 5 IU/mL, R2 + E50 = romiplostim 2 ng/mL+ rHuEPO 50 IU/mL, R2 + E100 = romiplostim 2 ng/mL+ rHuEPO 100 IU/mL, R2 + E200 = romiplostim 2 ng/mL + rHuEPO 200 IU/mL. Data are expressed as the mean ± standard deviation (*n* = 3).

To further elucidate the roles of rHuEPO and romiplostim in BM by evaluating their effect on HSPCs, we collected HSPCs from rat BM and cultured *in vitro* (Figure 5A). As shown in Figure 5B, the absolute numbers of HSPCs increased with the increasing concentration of romiplostim, which indicated that romiplostim could stimulate the proliferation of HSPCs. The CFU assays were performed to examine the differentiation of HSPCs with the addition of rHuEPO and romiplostim. It can be seen that the number of total BFU-E and CFU-E increased (Figure 5C), whereas the number of CFU-MK decreased (Figure 5D) with the increasing concentration of rHuEPO. Collectively, these *in vitro* results indicated that romiplostim expands HSPCs while rHuEPO promotes erythropoiesis and inhibits megakaryopoiesis indirectly through lineage competition.

### Combination Treatment Exhibits No Stimulatory Effects on Tumor Growth and Metastasis *In Vivo*

**Figure 6:**
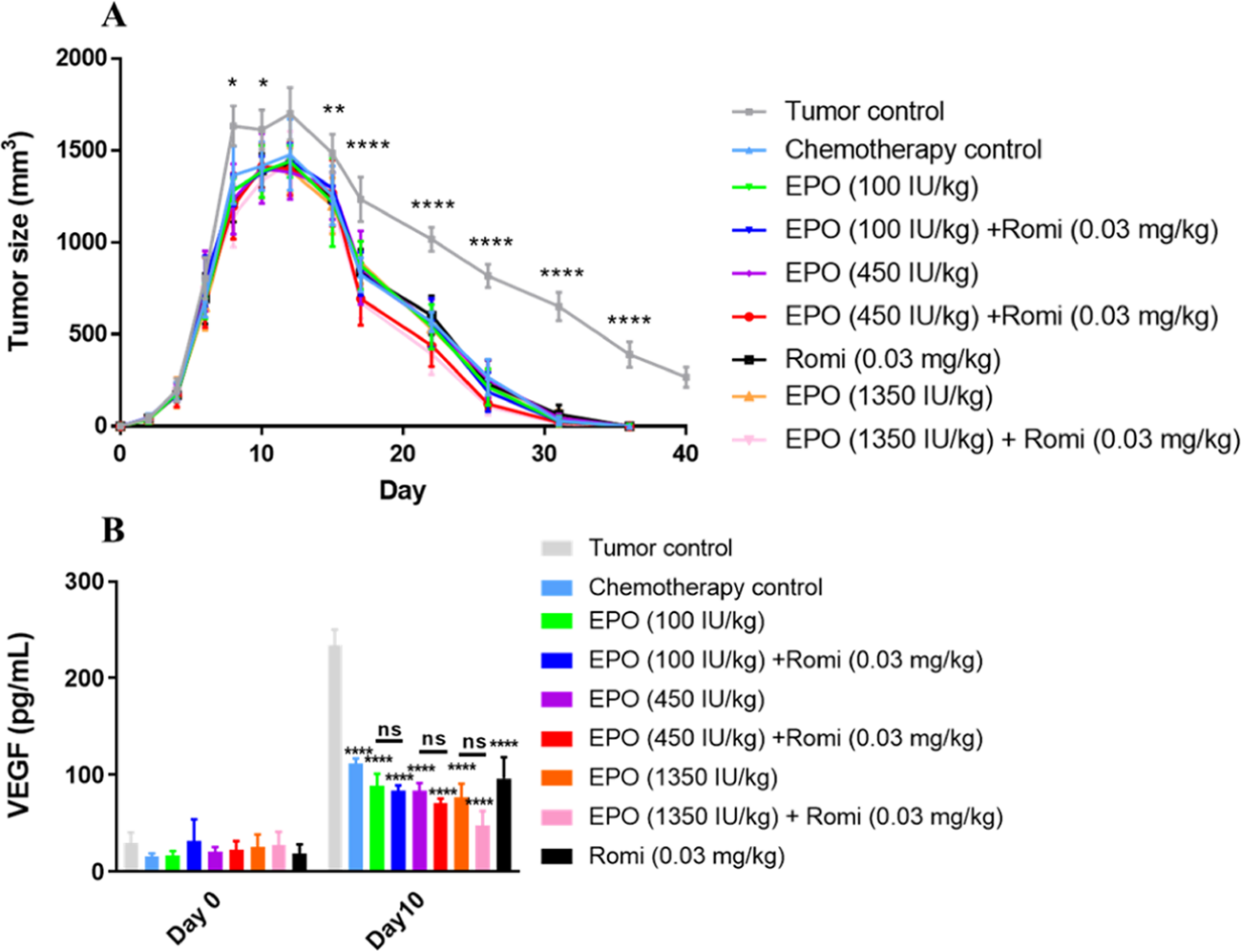
RHuEPO and/or romiplostim do not stimulate tumor growth *in vivo*. (A) Time course of mammary tumor volume progression in treated rats. (B) VEGF concentration in the serum. Data were expressed as mean ± standard deviation (*n* = 6). The symbols above the tumor size lines indicate days of statistically significant differences between the following tumor control group and the corresponding chemotherapy control group, *p < 0.05, **p < 0.01, ****p < 0.0001, two-way ANOVA with Dunnett’s multiple comparisons test for both tumor size and VEGF.

One concern of using rHuEPO and romiplostim to treat CIAT is the possibility of promotion of tumor growth and metastasis.^1,12^ However, some studies have shown that the administration of rHuEPO and TPO analogues is unlikely to stimulate tumor growth.^1,15,35−37^ Instead, rHuEPO could improve the accumulation and therapeutic effects of anticancer drugs.^38,39^ To investigate whether rHuEPO and romiplostim combination therapy concomitant chemotherapy could stimulate tumor growth and metastasis, the tumor volume, and the concentration of VEGF, a potent and specific angiogenic factor that is correlated with tumor growth,^40^ were measured. As shown in Figure 6A, progressive tumor growth was observed in the tumor control group from day 2 to day 12, and a decline in tumor volume was noted after day 12 in the tumor control group, which continued to day 40. This was consistent with previous studies and can be explained by an alloimmune response against the implanted tumors.^20,41,42^ After treatment with carboplatin on day 4, a significantly delayed tumor growth was observed compared to the tumor control rats, and the tumor volume decreased faster with a sharp downward shift of the tumor growth curves. And the tumor disappeared on day 36. Importantly, the rHuEPO and romiplostim monotherapy and combination therapy groups exhibit no stimulatory effects on tumor growth compared to the chemotherapy control group. The level of VEGF correlated with the tumor volume change (Figure 6B). The concentration of VEGF in tumor control rats increased significantly when tumor size reached a peak value, whereas the concentration of VEGF in carboplatin-treated rats decreased significantly compared with the tumor control rats, and neither rHuEPO and romiplostim monotherapy nor combination treatment increased VEGF levels in vivo. These results demonstrated that the combination treatment exhibited no stimulatory effects on tumor growth *in vivo*.

### PK-PD Modeling Provides Mechanistic Insights Regarding rHuEPO and Romiplostim Combination Therapy on CIAT

**Figure 7:**
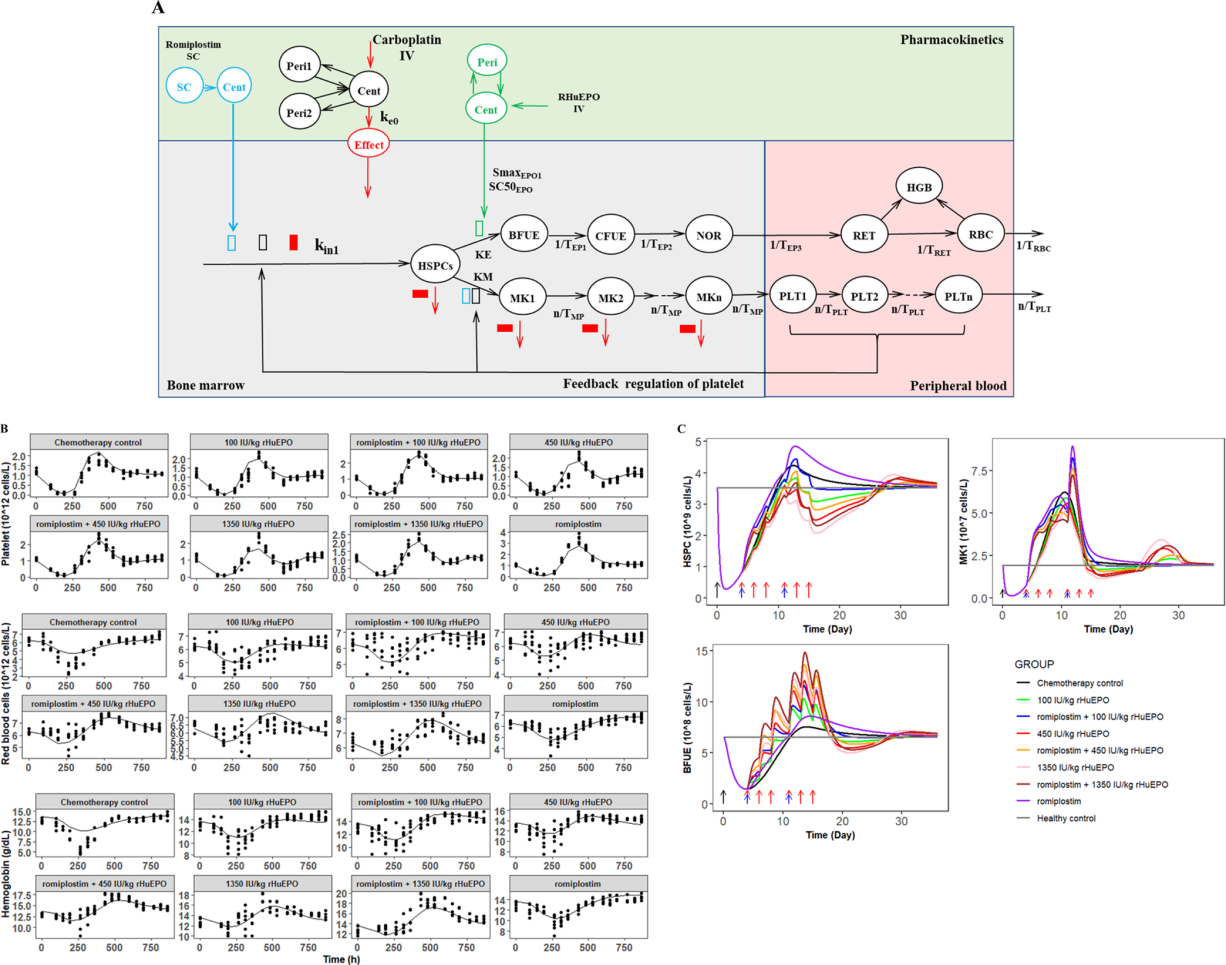
(A) Schematic diagrams of the proposed PK/PD model for the effects of carboplatin, rHuEPO, and romiplostim on red blood cells and platelet production. The open rectangle indicates the stimulatory effects of rHuEPO (green), romiplostim (black), and platelet feedback regulation (blue). The solid rectangle indicates the inhibitory effect of carboplatin (red). The other symbols, processes, and model operation are explained in the Materials and Methods section. (B) Platelet (top), red blood cells (middle), and hemoglobin (bottom) time course profile in different groups. The solid lines represent the median of the model predictions, and the dots represent the observed data. (C) Model-based simulations of HSPC, MK1, and BFUE. The arrows represent the dosing event of carboplatin (black), rHuEPO (red), and romiplostim (blue).

To quantify the effects of rHuEPO and romiplostim on CIAT under different treatments, a mechanism-based PK-PD model was established according to the data presented above (Figure 7A). In the model, carboplatin inhibits the proliferation and differentiation rate of HSPCs and induces apoptosis of MK. Correspondingly, romiplostim stimulates the proliferation of HSPCs and their differentiation into MK to increase platelet production but rHuEPO recruits HSPCs into the erythroid lineage to promote erythropoiesis. This model was adapted from our previous publication and based on our present study results.

The PK profiles of carboplatin, rHuEPO, and romiplostim were reasonably characterized by the proposed PK models (Figures S2 and S3). The PK parameters estimated for carboplatin, rHuEPO, and romiplostim are shown in Table S1. Due to the lack of iv PK data, the estimates of volume and clearance for ROM are apparent (V_7_ and CL_R_). Following the PK model development, the typical PK parameters obtained from the PK modeling were used to drive the PD effect.

Standard goodness-of-fit diagnostic plots showed no overall bias (Figure S4), suggesting an appropriate characterization of the PD data during treatment with rHuEPO and romiplostim as monotherapy and combination therapy by the PK-PD model. Figure 7B shows simulation results for the platelet, RBC, and Hgb responses. The predicted and observed PD values were in good agreement for all treatment groups, indicating that the model reasonably described the PD response of both monotherapy and the combination of rHuEPO and romiplostim in rats with CIAT.

The PD parameter estimates are given in Table 1. In general, the estimated lineage-related parameters, including RBC_0_, PLT_0_, *T*_RET_, MCH, *T*_MP_, and *T*_PLT_ were close to the physiologic values.^17^ The estimated RBC life span is 297.3 h and is shorter than the literature value of 1440 h. This is reasonable because cancer and treatments may shorten its life span. An alternative Imax model for carboplatin effect was initially tested, but the model was unstable and the parameters could not be precisely estimated. Accordingly, we simplified the model to the current linear function. All PD parameters were estimated with reasonable precision with RSE% less than 41%.

**Table 1:** Model Estimates of the PD Parameters Together with Their Relative Standard Errors (RSE)^a^

parameters (units)	description	estimate	%RSE
kill (mL/μg)	slope of carboplatin effect concentration for linear inhibition	0.06174	22.8
RBC_0_ (×10^12^ cells/L)	baseline RBCs concentration	6.343	1.308
*T*_RET_ (h)	mean residence time for RETs	35.13	9.669
*T*_RBC_ (h)	mean residence time for mature RBCs	297.3	13.72
PLT_0_ (×10^12^ cells/L)	baseline platelets in blood	1.009	2.064
*T*_MP_ (h)	mean life span of megakaryocyte cells	106	6.087
*T*_PLT_ (h)	mean life span of platelets	139.8	5.423
KE (×10^–4^/h)	first-order rate constant of HSPCs differentiate into BFU-E	52.94	16.02
MCH (pg/cell)	mean corpuscular Hgb	21.75	0.9106
Smax_RM1_	slope of romiplostim serum concentration on HSPCs	0.07491	10.7
Smax_RM2_	slope of romiplostim serum concentration on the differentiation of HSPCs into MK	0.08929	15.88
Smax_PLT1_	slope of platelet regulation on HSPCs	1.963	16.24
Smax_PLT2_	slope of platelet regulation on the differentiation of HSPCs into MK	2.768	15.95
Smax_EPO_	maximal stimulus of rHuEPO on HSPCs	2.784	19.78
SC50_E_ (mIU/mL)	the concentrations of rHuEPO that induce a half-maximum effect	66.08	40.56
*K*_e0_ (1/h)	equilibration rate constant	0.01669	20.05
σ_PLT1_	proportional error of platelets	0.1657	5.238
σ_PLT2_	additive error of platelets	0.1103	5.558
σ_RBC_	additive error of RBC	0.5616	5.208
σ_HGB_	additive error of HGB	1.177	6.137

a Notes: The PK parameters were fixed at their estimated values.

The simulated profiles of HSPCs, BFUE, and MK1 in different groups are shown in Figure 7C. The simulation results showed that the HSPCs decreased significantly after chemotherapy and recovered faster after romiplostim treatment. However, treatment of rHuEPO with or without romiplostim will inhibit the recovery of HSPCs because rHuEPO drives its differentiation into the erythroid lineage to promote erythropoiesis. For MK1, rHuEPO monotherapy will inhibit its recovery due to the HSPC competition mechanism. For BFUE, romiplostim monotherapy only promotes its production slightly, while the rHuEPO and romiplostim combination therapy could promote its production synergistically. These results are consistent with the PD results.

In summary, this model was capable of describing the PD response and provides mechanistic insights regarding rHuEPO and romiplostim monotherapy and combination therapy in rats with CIAT, which could serve as a valuable tool to inform the optimal dosing regimen of the combination therapy by simulating the expected hemoglobin and platelet responses in CIAT.

## Discussion

According to the estimates, the rising global cancer burden will increase the requirement for first-course chemotherapy by 53% between 2018 and 2040.^43^ However, cancer patients undergoing chemotherapy usually encounter CIAT, a major contributing factor to decreased quality of life and shortened survival.^10^ Currently, lineage-specific supportive care interventions, such as hematopoietic growth factors and blood transfusions, are still major management of CIAT, but these methods mainly focus on a single lineage, which manages CIA or CIT, respectively. The effective treatment of CIAT simultaneously may provide additional months or years of disease stability that likely translates into improved survival, which remains an important unmet need.^1,10^

In this study, we investigated a novel combination therapy of rHuEPO and romiplostim for treating CIAT simultaneously in cancer rats. The rationale for testing this innovative treatment strategy was based on our recent findings showing that romiplostim could synergize with rHuEPO to promote RBC production while EPO inhibited platelet production in a dose-dependent manner to reduce the risk of thrombocytosis., Recent reports also demonstrated the safety and efficacy of EPO-RA darbepoetin alfa and romiplostim for CIAT and radiation-induced myelosuppression in patients, which could achieve remission in the absence of blood product transfusions.^44−46^ In one case report, a patient with myelodysplastic syndrome received darbepoetin and concomitant usage of romiplostim.^46^ The results were consistent with our hypothesis that romiplostim was suggested to stimulate the erythroid response in addition to the effect of darbepoetin and the reduced darbepoetin dosage. Meanwhile, the platelet density did not increase during the combination usage of darbepoetin and romiplostim, supporting the MK-inhibiting effect of darbepoetin.

The primary safety concerns with the use of hematopoietic growth factors to manage CIAT are the possibility for the promotion of tumor growth and metastasis.^47^ It has been reported that LA-7 cells, a mammary adenocarcinoma cell line isolated from 7,12-dimethylbenanthracene-induced breast cancer rats,^48^ express EPO receptors.^33^ Moreover, the strong tumorigenic properties of the LA-7 cells could develop orthotopic solid mammary gland tumors in immunocompetent rats in a relatively short duration,^20^ which made the LA-7 model ideally suited for testing the effects of rHuEPO and romiplostim on tumor growth and metastasis both *in vitro* and *in vivo*. Therefore, our investigation began by studying the effects of rHuEPO and romiplostim on the growth and invasion of LA-7 *in vitro*. The results suggest that rHuEPO and romiplostim exhibit no stimulatory effects on the growth and invasion of LA-7 cells (Figure S1). The tumor volume and the level of VEGF in the serum were also detected in the LA-7-induced orthotopic rat model. There were no differences in tumor volume or VEGF level among the rHuEPO and romiplostim-treated groups and the chemotherapy control group in this model (Figure 6). Corroborating findings from *in vitro*, *in vivo* results further indicated that neither rHuEPO and romiplostim monotherapy nor combination therapy stimulate tumor growth and metastasis.

The advantages of the combination therapy compared with monotherapy are shown in Figure 2. For rHuEPO monotherapy, despite the gradual recovery of RBCs and Hgb, intensive rHuEPO treatment inhibited platelet production and increased the risk of secondary thrombocytopenia. For romiplostim monotherapy, the platelet increased sharply, which raised the risk of thrombocytosis. Notably, we can see that the rHuEPO and romiplostim combination therapy promoted RBC production synergistically, which could not only help to increase the erythropoietin-stimulating agents (ESAs) response rate but also help to reduce the rHuEPO dose requirement to avoid potential off-target effects. Moreover, in the combination therapy groups, the platelet rebound was inhibited compared with the romiplostim monotherapy group while the secondary thrombocytopenia was alleviated compared with the rHuEPO monotherapy groups. This diminished the risk of secondary thrombocytopenia and thrombocytosis simultaneously and addressed the safety concerns of the potential inducement of thromboembolism with the use of the combination therapy to manage CIAT. However, the effect of rHuEPO and romilostim on BM total cells (Figure 3C) and erythroid precursors (Figure 3D) is measured on day 40. At this time, all rHuEPO and romiplostim are cleared. Also, RBC, Hgb, and platelet are at the baseline level. The observed effect is not a direct effect of rHuEPO and romiplostim but results of some secondary mechanism perturbed by these drugs, which need further study.

According to the aforementioned findings, a novel mechanism-based PK-PD model was developed to quantify the effects of rHuEPO and romiplostim on megakaryopoiesis and erythropoiesis in CIAT (Figure 7A). The present PK-PD model shares a basic physiological model structure adapted from the literature,^17,18,49^ which adequately described the observed PD data after administration of rHuEPO and/or romiplostim. The GOF plots (Figure S4) and simulation results (Figure 7B) were used as complementary methodologies to evaluate the proposed model for RBC and platelet counts and Hgb concentration, which qualified the proposed model and confirmed the predictive performance. Our simulation results are consistent with the PD results (Figure 7C), which further demonstrated that romiplostim could synergize with rHuEPO to promote erythropoiesis, while rHuEPO reduces the risk of thrombosis caused by the romiplostim-induced increase in the platelet count through HSPC competition. The established model provides mechanistic insights regarding rHuEPO and romiplostim combination therapy on CIAT, which could serve as a valuable tool to inform the optimal dosing regimen of the combination therapy.

As far as we know, this study is the first to systematically demonstrate the beneficial effects of using EPO-RA and TPO-RA combination therapy for treating CIAT simultaneously. Both rHuEPO and romiplostim are marketed drugs with clinically proven efficacy. The safety of the combination therapy, however, is an important consideration for the effective clinical translation of our findings. The dosing regimen selection is critical for this combination therapy to achieve the synergistic effect on erythropoiesis and to maintain platelets in the normal range simultaneously. Moreover, considering that the incidence of CIT varies considerably between different regimens and various patient populations,^1^ this combination therapy strategy should be individualized accordingly. For example, if patients exposed to chemotherapy have grade 3 or 4 thrombocytopenia, then EPO-RA administration should be reduced or delayed to avoid more severe thrombocytopenia. Currently, different formulations and generations of ESAs are available, including the short-acting epoetin and long-acting darbepoetin.^2^ For TPO-RAs, peptibody romiplostim and small molecules such as eltrombopag, avatrombopag, and lusutrombopag are routinely used for the management of various thrombocytopenic states.^1^ Romiplostim was used in this study due to the wealth of published CIT data for this agent. Furthermore, romiplostim has a wide titratable range (from 1 to 10 μg/kg) and may have greater potency than oral TPO-RAs.^50^ Different combination strategies and dosing regimens of ESAs and TPO-RAs may also prove to be useful in the treatment of CIAT and warrant further evaluation.

The standard of care for the management of CIAT is not clear to date, especially with no approved agents available to manage CIT currently and with the controversies against the routine use of ESAs. Based on our findings presented here, we conclude that rHuEPO and romiplostim combination therapy can treat CIAT simultaneously while minimizing the risk of thrombosis and not stimulating tumor growth. These data may serve as a foundation for future clinical trials of this novel combination strategy in treating anemia and thrombocytopenia simultaneously in cancer patients receiving chemotherapy.

## Supporting Information Available

Additional information about PK-PD model estimates of the PK parameters (Table S1); *in vitro* study results (Figure S1); general goodness of fit of the final PK models (Figure S2); visual predictive check for the final PK models (Figure S3); and general goodness of fit of the final PD models (Figure S4) (PDF)
