# Supplementary material for: Novel Combination of Erythropoietin and Romiplostim to Treat Chemotherapy-Induced Anemia and Thrombocytopenia via Pharmacodynamic Interaction on Hematopoietic Stem and Progenitor Cells
Source: ACS Pharmacol Transl Sci. 2023 Nov 15;6(12):1884–97. doi: 10.1021/acsptsci.3c00194 (PMC10714423; doi:10.1021/acsptsci.3c00194)
Supplement: Supplementary file 1 — pt3c00194_si_001.pdf [file pt3c00194_si_001.pdf]

Supporting Information

A novel combination of erythropoietin and romiplostim to treat chemotherapy-induced anemia and thrombocytopenia via pharmacodynamic interaction on hematopoietic stem and progenitor cells

Xiaoqing Fan[1], Wojciech Krzyzanski[2], Raymond S. M. Wong[3], Dongyang Liu[4*], Xiaoyu Yan[1]*

[1]School of Pharmacy, Faculty of Medicine, The Chinese University of Hong Kong, Shatin, Hong Kong, China SAR

[2]Department of Pharmaceutical Sciences, The State University of New York at Buffalo, Buffalo, NY, USA

[3]Division of Hematology, Department of Medicine and Therapeutics, Faculty of Medicine, The Chinese University of Hong Kong, Shatin, Hong Kong, China SAR

[4]Drug Clinical Trial Center, Peking University Third Hospital, Beijing, China

*** Corresponding author 1: Xiaoyu Yan**

Tel: +852-34935012; **Fax:** +852-26035295.

Email address: xiaoyuyan@cuhk.edu.hk

*** Corresponding author 2: Dongyang Liu**

Email address: liudongyang@vip.sina.com

Table S1. Model estimates of the PK parameters together with their relative standard errors.

Parameters (Units)	Description	Estimate	%RSE
**CL_E (mL/h)**	Clearance of rHuEPO	9.696	1.644
**Q_E (mL/h)**	Tissue distribution clearance of rHuEPO	2.959	1.275
**V_4 (mL)**	Volume of distribution of the central compartment of rHuEPO	44.38	1.275
**V_5 (mL)**	Volume of distribution of the peripheral compartment of rHuEPO	11.97	3.596
**σ of rHuEPO**	Additive error in logarithmic domain	0.2624	11.79
**CL_R (mL/h)**	Clearance of romiplostim	29.28	2.235
**V_7 (mL)**	Volume of distribution of the central compartment of romiplostim	634.9	2.95
**Ka (1/h)**	Absorption rate of romiplostim	0.1874	19.14
**σ of romiplostim**	Proportional error in logarithmic domain	0.4048	15.86
**$K_{el(CAR)}$ (1/h)**	Elimination rate constant of carboplatin	2.504	4.907
**V_{Car} (mL)**	Volume of distribution of the central compartment of carboplatin	158.8	3.879
**$K_{CP1(CAR)}$ (1/h)**	Intercompartment rate constant of carboplatin	0.06625	95.59
**$K_{PC1(CAR)}$ (1/h)**	Intercompartment rate constant of carboplatin	0.5182	70.8
**$K_{CP2(CAR)}$ (1/h)**	Intercompartment rate constant of carboplatin	3.556	1.683
**$K_{PC1(CAR)}$ (1/h)**	Intercompartment rate constant of carboplatin	4.059	0.9145
**$\sigma_{additive}$ of carboplatin**	Additive error	0.08072	14.2
**σ_{prop} of carboplatin**	Proportional error	0.07049	18.74

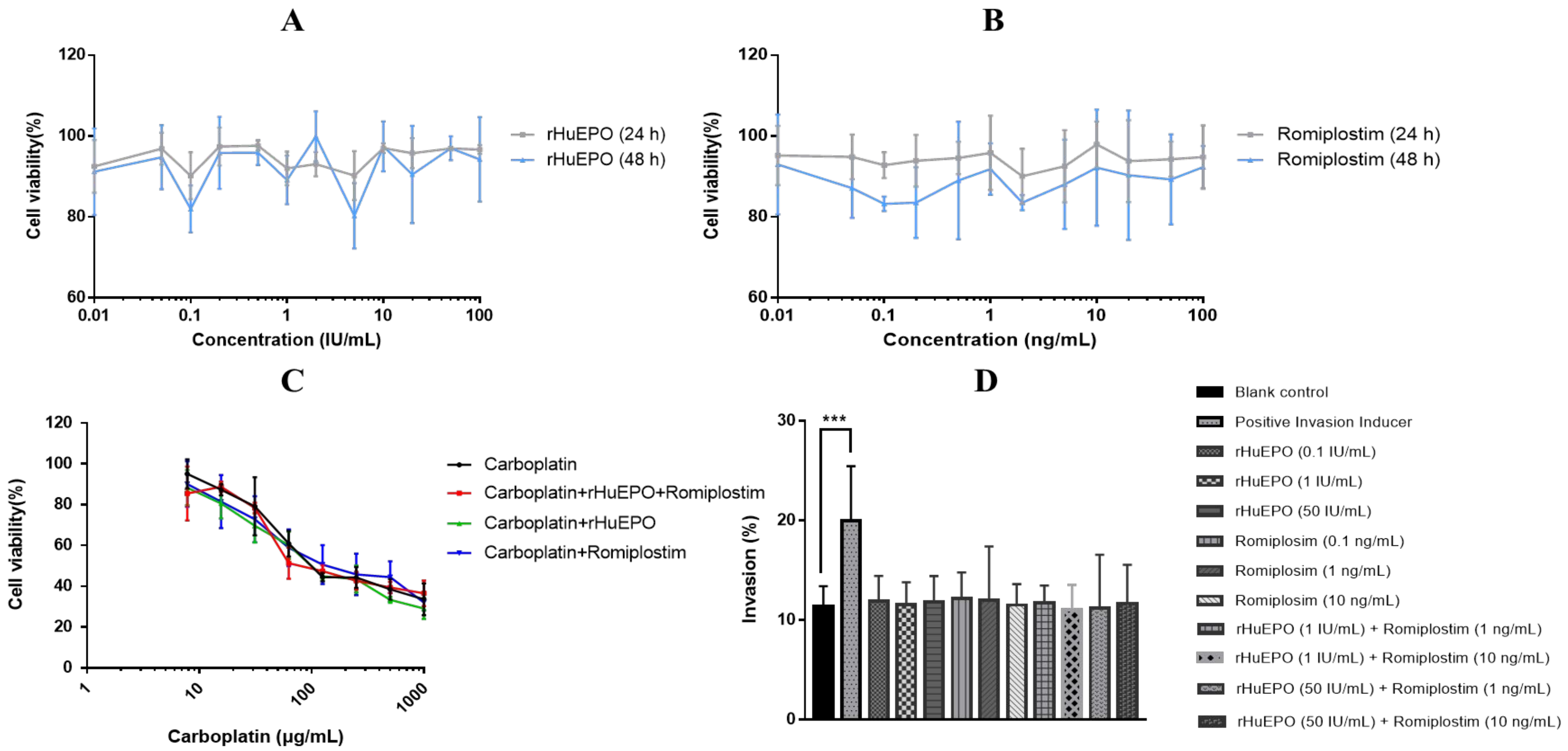

Figure S1. RHuEPO and/or romiplostim do not stimulate LA-7 breast cancer cells growth and invasion *in vitro*. (A) RHuEPO concentration-response (cell viability) curves; (B) Romiplostim concentration-response (cell viability) curves; (C) Carboplatin concentration-response (cell viability) curves in the absence or in the presence of rHuEPO (1 IU/mL), romiplostim (4 ng/mL), rHuEPO (1 IU/mL) + romiplostim (4 ng/mL) for 48 h; (D) The invasion of LA-7 cells, the cell invasion percentage was defined as the ratio of cells in the lower chamber to the total cells added to the top chamber. Data were expressed as the mean ± standard deviation of six independent experiments (n=6). ***$p < 0.001$, one-way ANOVA with Dunnett's multiple comparisons test.

A

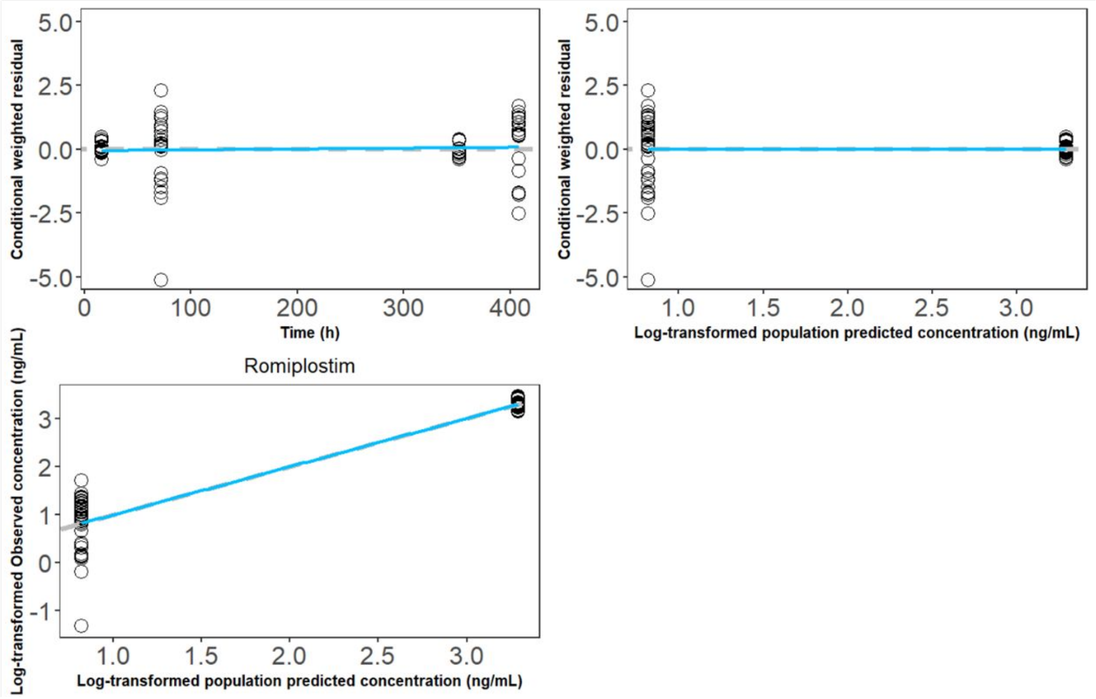

Conditional weighted residual
Time (h)
Log-transformed population predicted concentration (ng/mL)
Romiplostim
Log-transformed Observed concentration (ng/mL)

B

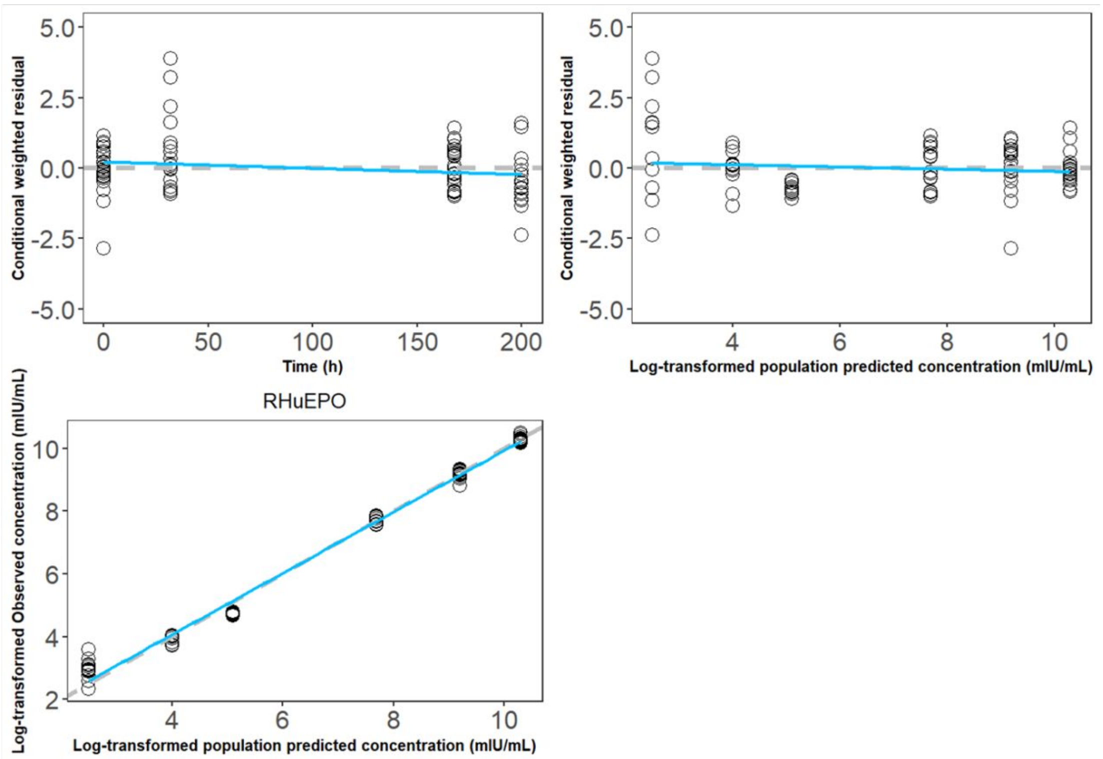

Conditional weighted residual
Time (h)
Log-transformed population predicted concentration (mIU/mL)
RHuEPO
Log-transformed Observed concentration (mIU/mL)

C

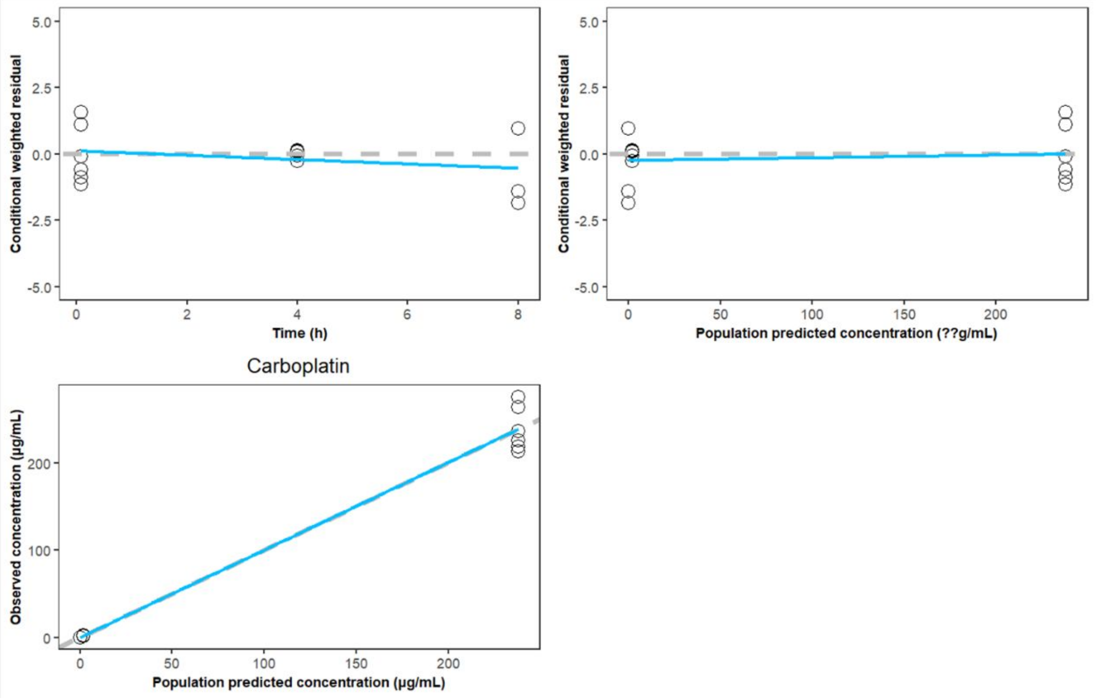

Figure S2. General goodness-of-fit of the final model for romiplostim (A), rHuEPO (B), and carboplatin (C). The top panels of (A), (B) and (C) present the conditional weighted residual (CWRES) vs. time (left) and population predictions (right), respectively. The bottom panels of (A), (B) and (C) present the observed data vs. population predictions. The blue lines are the loess smooth lines. The gray diagonal (top panels) and horizontal (bottom panels) lines are the identity and zero lines, respectively.

A

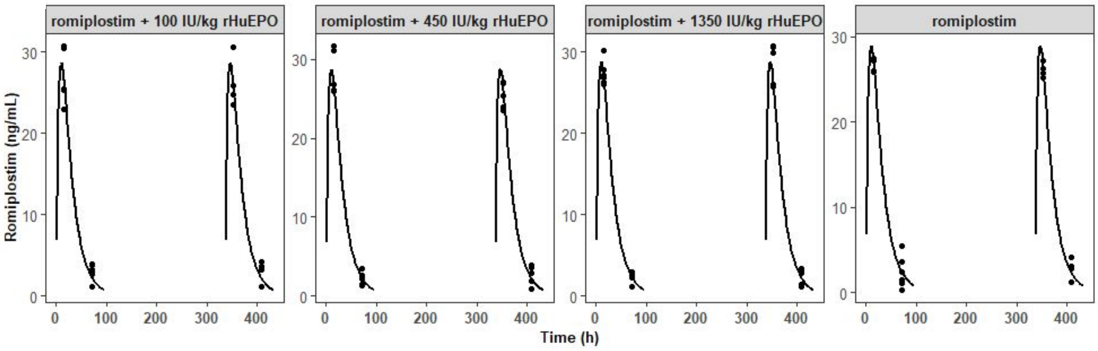

B

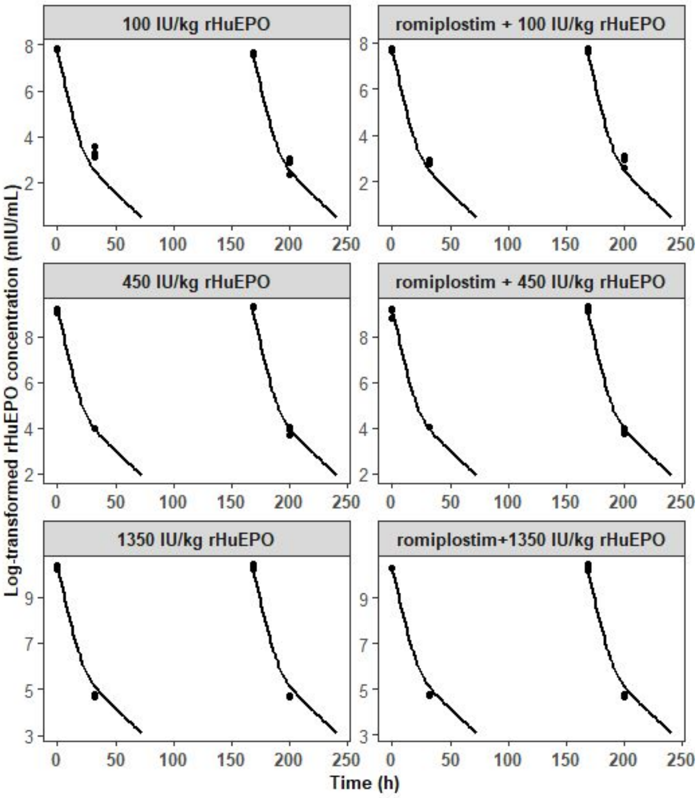

C

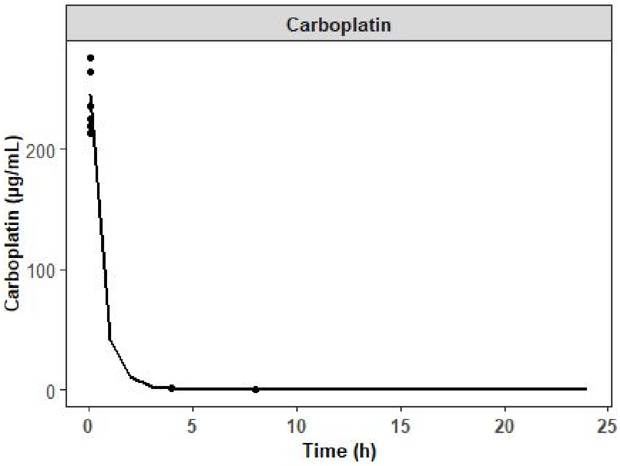

Figure S3. Visual predictive check for the romiplostim (A), rHuEPO (B), and carboplatin (C) in different groups. The solid lines represent the median of the model predictions, and the dots

represent the observed data.

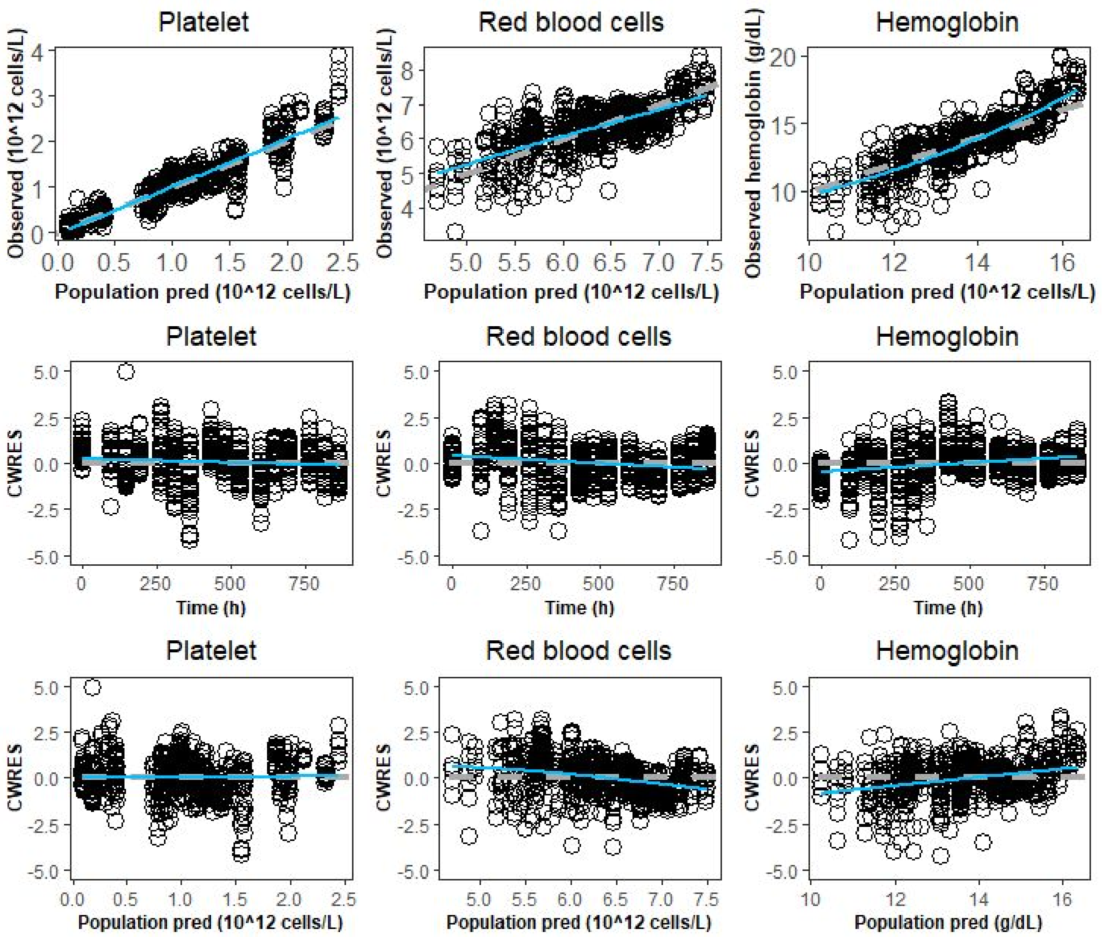

Figure S4. General goodness-of-fit of the final PD model including platelet (PLT, left panels), red blood cells (RBCs, middle panels), and hemoglobin (HgB, right panels). Following the up-to-bottom order, the panels present the observed data vs. population predictions, conditional weighted residual (CWRES) vs. time, and CWRES vs. poplation predictions, respectively. The blue lines are the loess smooth lines. The gray diagonal and horizontal lines are the identity and zero lines, respectively.
